# An automated quasi-continuous capillary refill timing device

**DOI:** 10.1088/0967-3334/37/1/83

**Published:** 2015-12-07

**Authors:** L L Blaxter, D E Morris, J A Crowe, C Henry, S Hill, D Sharkey, H Vyas, B R Hayes-Gill

**Affiliations:** 1 Electrical Systems & Optics Research Division, Faculty of Engineering, University of Nottingham, University Park, Nottingham NG7 2RD, UK; 2 Academic Child Health, School of Medicine, E Floor East Block, Queen’s Medical Centre, Nottingham NG7 2UH, UK

**Keywords:** capillary refill time, automation, sensor, monitoring

## Abstract

Capillary refill time (CRT) is a simple means of cardiovascular assessment which is widely used in clinical care. Currently, CRT is measured through manual assessment of the time taken for skin tone to return to normal colour following blanching of the skin surface. There is evidence to suggest that manually assessed CRT is subject to bias from ambient light conditions, a lack of standardisation of both blanching time and manually applied pressure, subjectiveness of return to normal colour, and variability in the manual assessment of time. We present a novel automated system for CRT measurement, incorporating three components: a non-invasive adhesive sensor incorporating a pneumatic actuator, a diffuse multi-wavelength reflectance measurement device, and a temperature sensor; a battery operated datalogger unit containing a self contained pneumatic supply; and PC based data analysis software for the extraction of refill time, patient skin surface temperature, and sensor signal quality.

Through standardisation of the test, it is hoped that some of the shortcomings of manual CRT can be overcome. In addition, an automated system will facilitate easier integration of CRT into electronic record keeping and clinical monitoring or scoring systems, as well as reducing demands on clinicians.

Summary analysis of volunteer (*n* = 30) automated CRT datasets are presented, from 15 healthy adults and 15 healthy children (aged from 5 to 15 years), as their arms were cooled from ambient temperature to 5°C. A more detailed analysis of two typical datasets is also presented, demonstrating that the response of automated CRT to cooling matches that of previously published studies.

## 1. Introduction

Capillary refill time (CRT), is a measure of the time taken for skin colour to return to normal following the blanching of blood via externally applied pressure. When measured manually the blanching pressure is applied using a finger or thumb with the time for the return of skin tone estimated by observation.

Manually applied CRT has been found to be of use in the assessment of perfusion (Osborn *et al* 2004), dehydration (Saavedra *et al* 1991) and shock (Pamba and Maitland 2004). However, the optimum application pressure and duration are currently poorly standardised, even though there is evidence that these factors can significantly influence the measured time (Pickard *et al* 2011). Also, environmental conditions such as variability in lighting and temperature both influence the assessment of CRT (Gorelick *et al* 1993, Brown *et al* 1994), and manual time measurement introduces observer variability.

Consequently, an automated CRT monitor incorporating: a means of controlled pressure application and release; an optical reflectance measurement to capture the refill profile; with data processing to then extract the CRT, could result in large reductions in the variance between measurements. If these refill data were available electronically, this would allow simpler and less error prone integration into electronic record keeping and/or clinical assessment systems. In addition, measurements could be made quasi-continuously, since when performed manually, the sampling rate will vary from tens of minutes to hours.

Although semi-automated CRT measurement has been demonstrated with the refill monitored via the photo-plethysmogram using blue light (Kviesis-Kipge *et al* 2009) and video analysis (Shavit *et al* 2006), a fully automated device that can be attached to the patient and then left to make regular measurements has not been clinically demonstrated. US Patent 8,082,017 Messerges and Hutchinson (2011) describes a modified pulse oximetry sensor incorporating an actuator to allow capillary refill to be applied to the finger tip, and US Patent App. 13/518,452 Bezzerides and Neuman (2010) describes a similar scheme utilising a pneumatic pressure applicator. However, a transmission mode device such as this would not be practical in some populations such as small children who would not keep the device on in the described configuration.

This paper introduces a new instrument that uses pneumatics to apply pressure to blanch the skin, and optical assessment of diffuse reflectance to monitor the refill signal. The CRT can then be determined at any site where a reflectance probe can be placed, a development that may be especially relevant to small and/or active children.

## 2. Overview

The earliest reported clinical use of CRT was as an indicator of dehydration in 1910 by Takayesu and Lozner (1910), but the authors made it clear that the test was already commonly known by that time. By 1940, Guedel (1940) were using capillary refill time measured on the forehead during surgery as an indicator of surgical shock. In 1947, Crismon and Fuhrman (1947) used the capillary refill effect to study the effects of frostbite in rabbits. Since the 1940s, capillary refill has become more widely used as a simple indicator for shock and dehydration.

Use of capillary refill as part of a numerical trauma score was first popularized by Champion in 1981 Champion *et al* (1981), who suggested applying pressure for 5 s, with a CRT of more than 2 seconds being indicative of shock. More recently trauma scores have been further developed, for example as the paediatric early warning system (PEWS) (Duncan *et al* 2006), where ‘the dynamic items (urine output, perfusion, pulse rate, and level of consciousness) could not be reliably abstracted and were not analyzed’. An automated capillary refill device may provide a reliable surrogate of perfusion, and thus allow a perfusion term to be included in the PEWS metric. Fleming *et al* present a systematic review of CRT studies in children, suggesting CRT >3 s as an indicator of abnormality (Fleming *et al* 2014).

However, use of the capillary refill metric in clinical practice is controversial. For example Baraff was highly critical of Champion’s work (Baraff 1993), noting the lack of comprehensive studies both into the nature of the capillary refill signal and into the inter versus intra group variability between healthy patient groups and groups suffering from e.g. shock. Schriger reached similar conclusions regarding use of a simple cut-off time for defining normal capillary refill (Schriger *et al* 1988).

An automated capillary refill device may allow some of these controversies to be resolved through standardisation of pressure application and release, and electronic measurement of diffuse reflectance, eliminating observer bias. Simultaneous recording of skin surface temperature may also allow environmental temperature induced changes to be compensated for (a technique described elsewhere (Bezzerides and Neuman 2010)).

### 2.1. Consideration of device functions

The core functionality required to make CRT measurements is to apply pressure to the skin to force out the blood in the underlying capillaries and then monitor the capillary refill after the pressure is released. As a fully automated capillary refill device has not been demonstrated previously in clinical practice, the functional details such a device should possess were unclear. Although the hardware discussed here is not intended to resemble a potential commercial device in all respects (for example aesthetics and enclosure design), serious consideration was given to design for manufacture and addressing the question of what design principles would be needed for a practical capillary device suitable for routine clinical use.

In existing clinical practice, CRT is usually taken from the nailbed or centrally on the sternum, with the sites on the forearm or knee being used in some cases (Shaw and Spandorfer 2005). With an automated device, standardisation of the application pressure is possible, so it may be feasible to extend the range of sites without unduly influencing the refill time measurement. It was determined that a compact reflection mode sensor, secured to the skin surface with adhesive was the most appropriate sensor format to allow use at a wide range of sites. This design addresses the disadvantages of other proposed systems, such as finger clip based designs (Bezzerides and Neuman 2010, Messerges and Hutchinson 2011).

Use of a disposable, low cost sensor in conjunction with a larger, bedside base unit was considered to be the form of a likely final design due to hygiene and practicality concerns. Although the need for a compact and extremely low cost single use sensor rules out many electromechanical pressure application methods, pneumatic devices are in widespread clinical use (for example blood pressure cuffs and pneumatic respiration sensors). For this reason it was decided that a pneumatic pressure application was most suitable, using a simple air bladder to apply pressure to blanch the skin surface and an arrangement of optodes to measure diffuse reflectivity.

The blanching pressure and application time are poorly standardised in manual capillary refill timing (Klupp and Keenan 2007, Pickard *et al* 2011). However, since it would be expected that an application pressure higher than systolic blood pressure would cause blanching, a pressure of 130 mmHg (17 kPa) was chosen for the applied pressure. The duration of pressure application is similarly ill defined, and so a time of 7 s was chosen, being longer than the 3–5 s normally used, and so allowing sufficient time to guarantee effective blanching.

## 3. Method

### 3.1. Sensor

**Figure 1:**
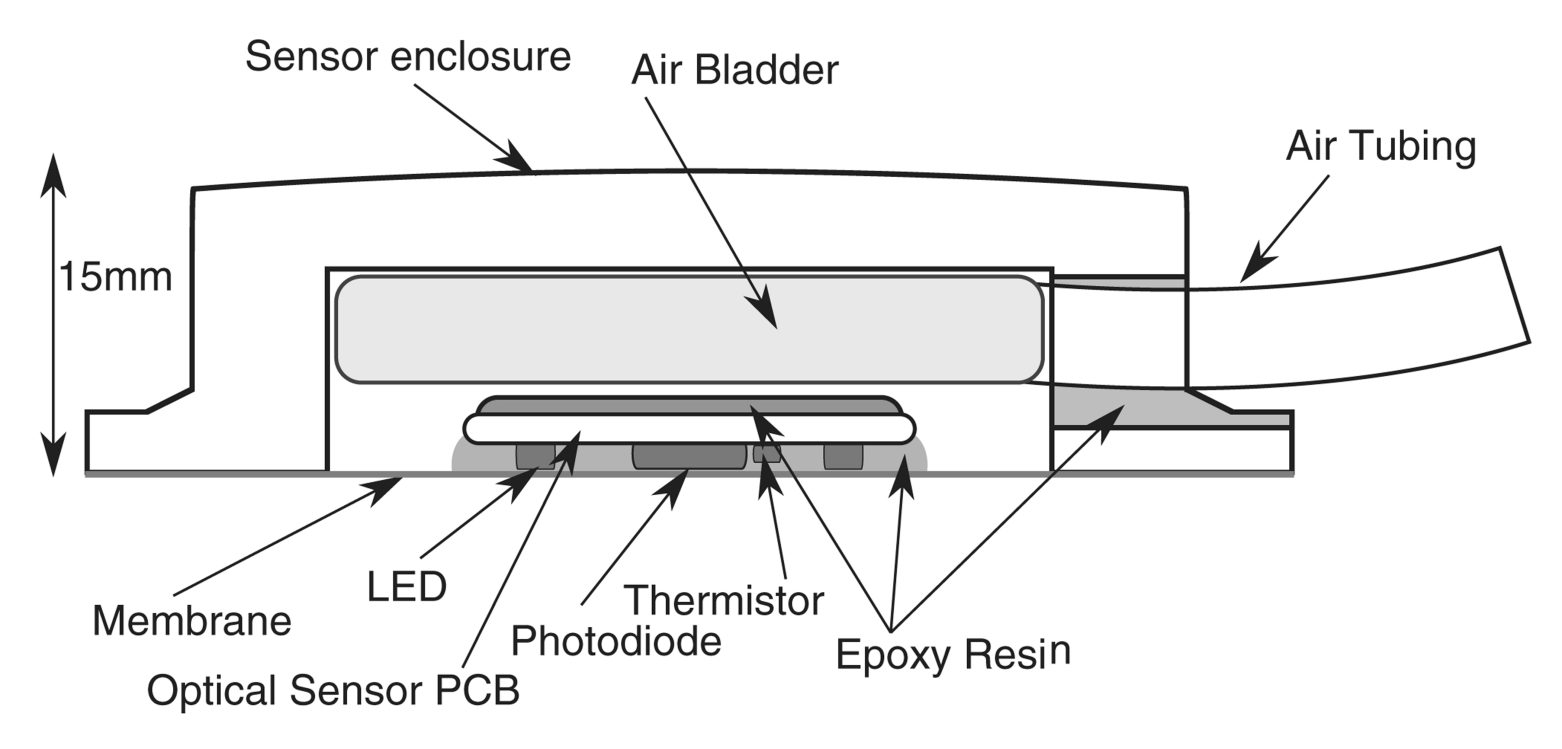
Cross sectional diagram of sensor construction. A thermistor was mounted to the skin facing side of the PCB, allowing approximate skin surface temperature measurement.

Figure 1 is a cross sectional diagram of the sensor construction. The outer enclosure is 42 mm square by 15 mm thick, and fabricated from ABS plastic. A folded size 1 (3 by 6 cm) blood pressure cuff (CRITIKON soft cuff, GE Healthcare, Little Chalfont, Buckinghamshire, United Kingdom) between the back of the PPG sensor and the enclosure allowed a pneumatic supply to be used to vary the application pressure. A photodiode (Vartec, VTB8440B) and ring of 6 LEDs emitting at 520 nm, 640 nm, and 950 nm (2 at each wavelength, 0603 size sourced from Osram and Kingbright) were separated by approximately 3 mm on a PCB made from 0.8 mm FR4 material. This PPG optode design is similar to that used by Grubb *et al* (2014). However, the addition of 640 nm and 950 nm to the green light design of Grubb *et al* potentially allows pulse oximetry measurements to be made with this device, and the effect of wavelength upon CRT data quality to be investigated. A thermistor (NCP15XH103F03RC, Murata, Kyoto, Japan) was mounted on the PCB to allow approximate skin surface temperature measurement. The lower membrane was formed from a biocompatible (ISO10993) polyurethane elastomer (EU95, Smith and Nephew extruded films, Gilberdyke, UK). The sensor hose assembly used Mogami 2754 shielded microphone cable (Marshall Electronics, CA, USA) passed through Tygon S-50-HL bio-compatible (ISO10993) PVC tubing (Saint-Gobain S.A., Paris, France), with the hose also forming the pneumatic supply line.

### 3.2. Pneumatic pressure application system

Small (maximum dimension ~5 cm, mass <100 g) diaphragm pumps capable of pumping to pressures in the range of 40 kPa or more with flow rates in the range of 1 L min^−1^are readily available and suitable for this application. Since these pumps are not reversible, a solenoid valve must be used to rapidly vent air from the system, releasing the applied pressure.

**Figure 2:**
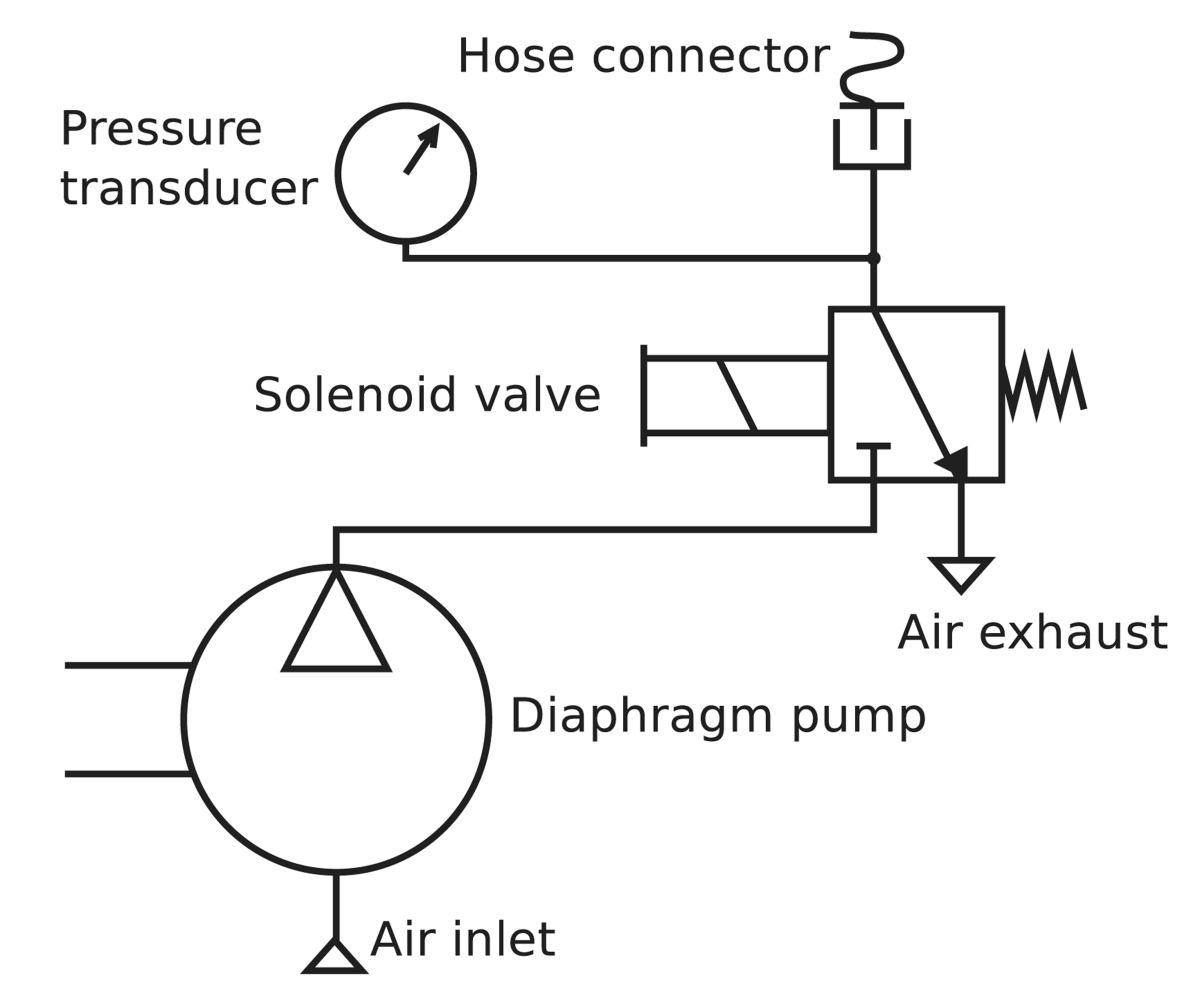
The pneumatic system designed for the CRT data-logger.

Figure 2 shows a schematic view of the pneumatic system, a design identical to that used in many commercial blood pressure meters, and comprising a diaphragm pump (Parker-Hanniffin corporation T5-1IC-03-1EEB, Ohio, USA), solenoid valve (912-000007-005), and a blood pressure cuff connector from an Omron M2 blood pressure monitor (Omron Corporation, Tokyo, Japan).

**Figure 3:**
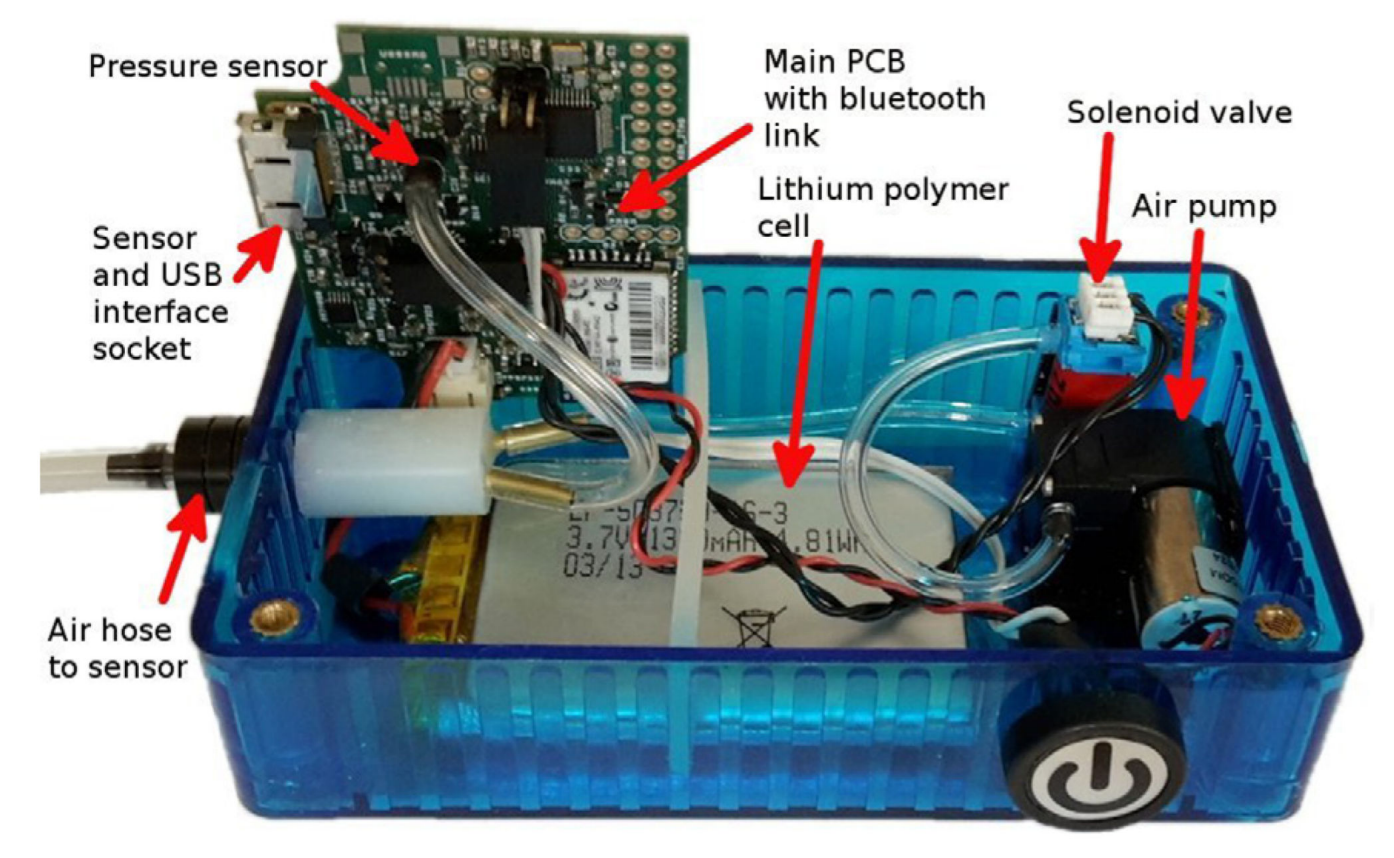
The CRT data-logger base unit, partially assembled view.

Figure 3 shows a top view of the partially assembled data-logger base unit. The Omron air hose connector can be seen below the main PCB (grey plastic).

### 3.3. Photodiode front end, DSP, and datalogger design

#### 3.3.1. Modulation technique

Almost all existing commercial PPG and pulse oximetry systems use clusters of light emitting diodes (LEDs), with different LEDs emitting at discrete wavelengths. This leads to a potential problem of isolating the detected signals from individual LEDs at the photo-detector. Time division and frequency division multiplexing are common solutions to this problem. Joseph *et al* describe a frequency division scheme applied to near infrared spectroscopy (Joseph *et al* 2006), and a typical implementation of time division can be seen in a pulse oximetry publication by Cohen and Wadsworth (1972).

Three factors were considered in an evaluation of modulation techniques: signal to noise ratio (thermal, shot, and 1/f noise), susceptibility to optical interference (e.g. light sources, compact fluorescent lights etc), and the impact of artefacts (this is especially relevant given that capillary refill studies involve inducing relatively sudden changes in tissue properties). These considerations were explored by Theodor *et al* (2013).

A frequency division scheme was adopted based on a consideration of amplifier 1/f noise (by adopting a frequency division scheme it is easier to operate above the 1/f corner frequency). It is also possible to minimise interference from ambient light sources such as compact fluorescent lights (which often contain electronic ballast modulating the light in the <5 kHz range). Additionally, for CRT use, the demodulated output data rate needs to be sufficiently fast that aliasing is minimized. Considering the typical time scale of capillary refills, with intensity transients over as little as 100 ms (Kviesis-Kipge *et al* 2009), this rate needs to be of order 100 Hz. However, given the transient nature of the refill, it seems likely that a high output data rate will not eliminate all aliasing. Time division multiplexing leads to mapping of artefacts more strongly to some channels than others, whereas frequency division gives rise to broadening of the modulation spectrum peaks, and cross-talk between channels. Cross-talk is a more preferable side-effect, so frequency division is optimal for mitigation of artefacts.

#### 3.3.2. Orthogonal frequency generation

Many micro-controllers include PWM (pulse width modulation) hardware, but these are only capable of generating orthogonal periods, not orthogonal frequencies. However, using PWM controller units with a ‘gate’ input allows a PWM output from one unit to control the time period for which a second unit is clocked from a central source. Unfortunately this leads to a small amount of jitter, giving cross-talk between the carriers.

**Figure 4:**
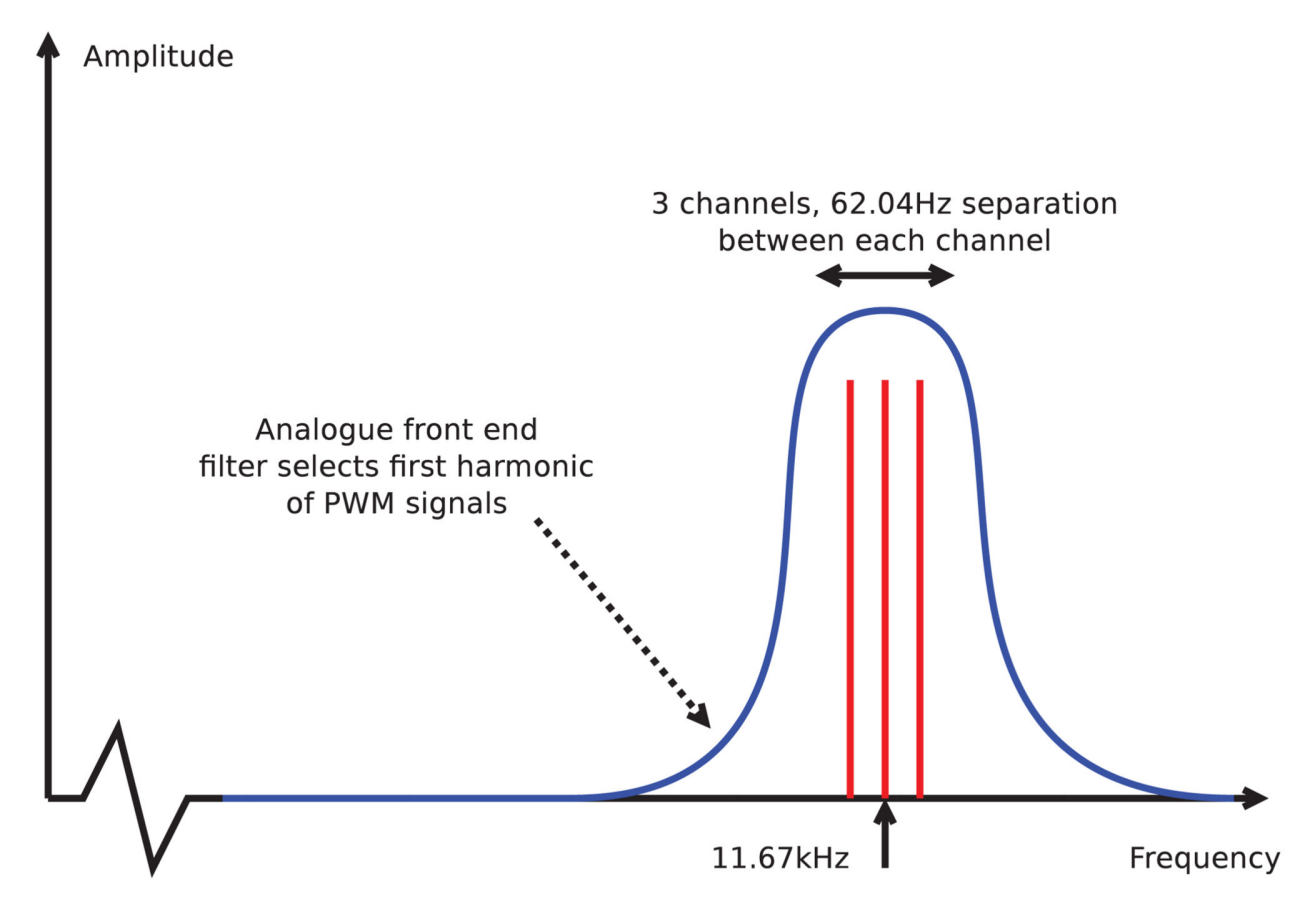
Schematic of the frequency division scheme adopted for use with the CRT datalogger device.

Since the extent of the jitter is affected by the precise topology of the gating system, an optimisation algorithm was employed to find the optimal configuration for a 3 channel system. The adopted scheme is shown in figure 4, with the precise choice of centre frequency and carrier spacing made based on the available clock tree configurations of the micro-controller, with the 62.04 Hz spacing and 11.67 kHz central carrier allowing simple integer multiplication and DFT to be used for carrier separation.

#### 3.3.3. Photodiode amplifier

Many articles have been published on the subject of low noise photodiode amplification, with the most common solution being a JFET voltage follower combined with a low voltage noise op-amp in a trans-impedance configuration. This configuration allows use of a low input referred voltage noise but high input current noise JFET op-amp (e.g. Texas Instruments LMH6624), but such a scheme leads to a relatively high current consumption and operating voltage of 5 V or more, suboptimal for a portable battery powered device. There are few low operating voltage op-amps with very low input voltage noise (<5 nV ${\sqrt{\mathrm{Hz}}}^{-1}$) and low input current noise.

**Figure 5:**
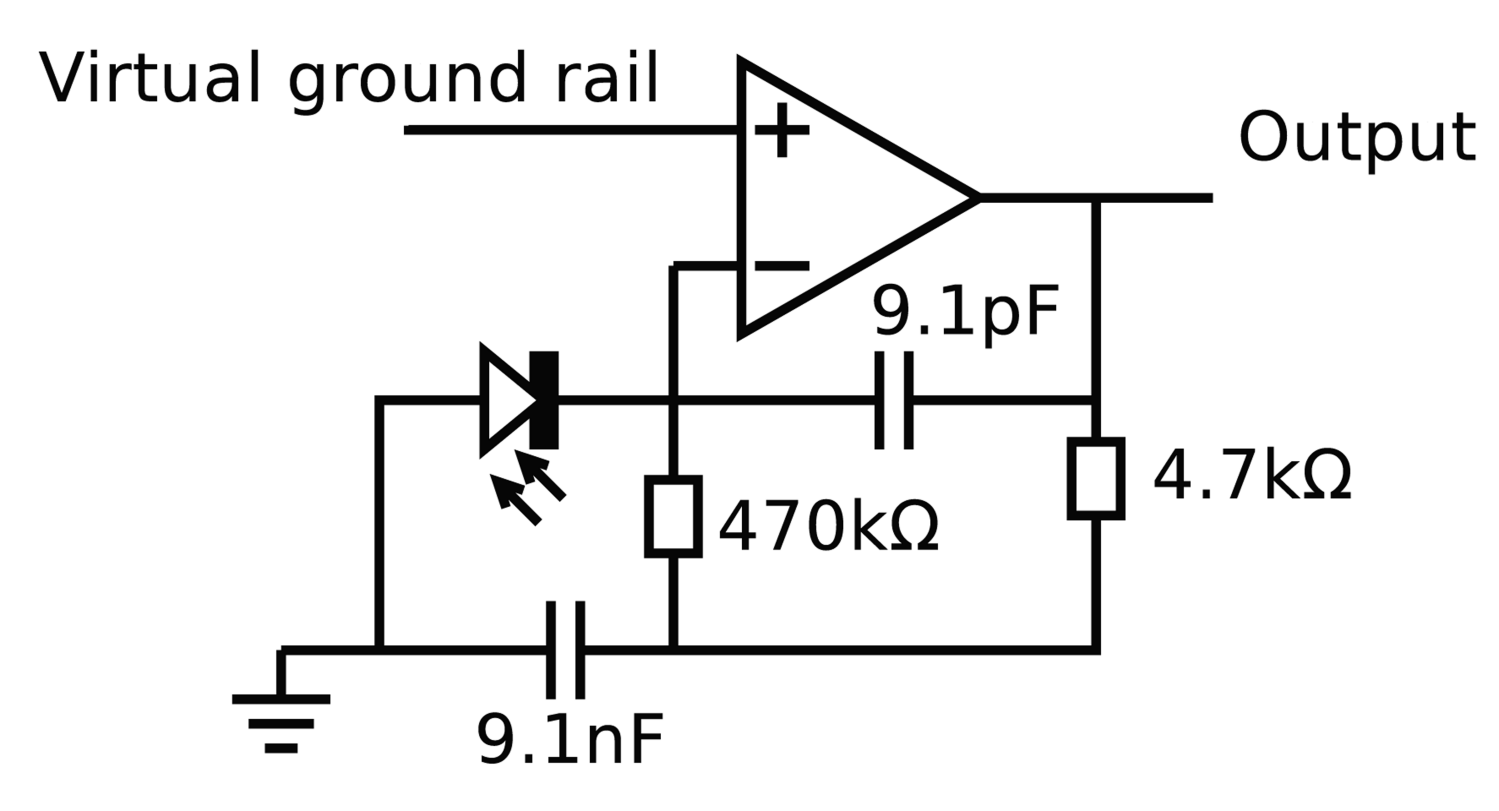
Single op-amp photodiode amplifier, designed using optimisation in SPICE package (virtual ground is held at 1.26 V).

However, it is only necessary to reduce amplifier noise to the optically limited shot noise floor. The worst case DC photocurrent was found to be of order 1 *μ*A with the sensor placed against the skin surface with a 20 mA mean LED current. This made a design based around a single op-amp feasible without significantly compromising the signal to noise ratio. In order to exclude as much wideband noise from the ADC as possible, a trans-impedance design incorporating a high *Q* filter based on a modified gyrator circuit was employed (figure 5). This design also enabled front end saturation to be detected directly by monitoring the output voltage, allowing LED brightness to be reduced when necessary.

The MCP6021 (MicroChip Inc.) op-amp was used due to its availability in a small 5 pin SOT23 package, low operating voltage, low input referred voltage noise, and low (30 pA typical) bias current. A virtual ground voltage of 1.26 V was found to be optimal under typical operating conditions (i.e. ambient light level), and a supply rail at 3.3 V. This resulted in a peak to peak photocurrent of approximately 500 nA, and mean of approximately 250 nA.

By modelling photocurrent shot noise, trans-impedance thermal current noise (in this case the 470 kΩ resistor), and the effect of voltage noise from the op-amp specification, the effective number of bits was calculated for the front end. The op-amp voltage noise (8.5 nV ${\sqrt{\mathrm{Hz}}}^{-1}$ at 11.67 kHz) was modelled as acting across the effective parallel capacitance of the photodiode and cable (200 pF from VTB8440B photodiode and 1m of Mogami 2754 miniature shielded cable). Opamp noise current density of $3\mathrm{fA}∕\sqrt{\mathrm{Hz}}$ is negligible compared to the other noise sources so was ignored. Assuming an RMS AC photocurrent of 177 nA (250 $\mathrm{nA}∕\sqrt{2}$):

(1) $$\mathrm{ENOB}=\frac{\ln\left(4.9\times 10^{5}∕\sqrt{\Delta f}\right)}{\ln\left(2\right)}=18.9-0.72\;\ln\left(\Delta f\right)$$

This is a high enough SNR to allow the small PPG cardiac synchronous intensity modulation to be accurately monitored (e.g. 17.2 bits, with Δ*f* bandwidth of 10 Hz).

**Figure 6:**
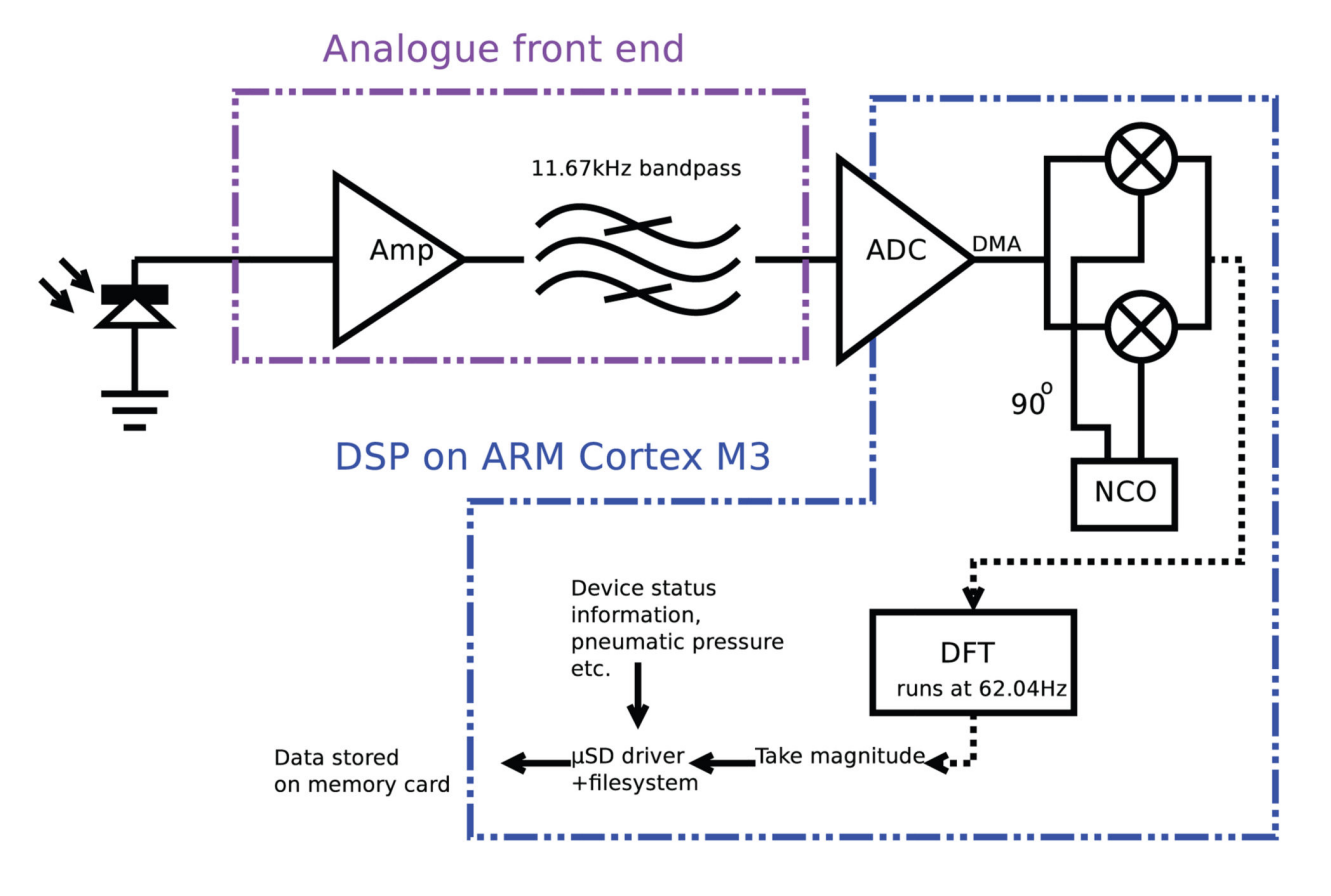
Schematic of the DSP processing scheme adopted for use with the CRT datalogger device.

Figure 6 shows the demodulation architecture designed to demodulate the 3 optical channels. ADC values are passed to RAM via DMA, before being read from an interrupt service routine. A complex (i.e. in-phase and quadrature) local oscillator at 11.67 kHz is used to downconvert the data to a complex baseband, and a DFT (with 1/62.04 s or 16.1 ms bin length) then separates the three optical carriers. Threaded routines running through an RTOS (Real Time Operating System) then process the data and store it. Pneumatic control and status code logging is carried out by other RTOS threads.

### 3.4. Control and datalogging system design

The datalogger was based around an STM32F103 micro-controller from ST-Microelectronics (Geneva, Switzerland). A Bluetooth module (RN-42, MicroChip Inc.) was attached via the microcontroller’s UART, allowing wireless data streaming and device firmware updates (using a bootloader).

A single 18 pin interface connector on the front face of the enclosure allowed PPG sensors to be connected to the device, or alternatively for the datalogger to be connected to a PC, allowing data download (using the mass storage device class), and charging of the logger’s 3.7 V lithium polymer cell (using 500 mA charging current). A microSD memory card formatted with a FAT32 filesystem was used to store logged data, and the real time clock peripheral built into the STM32 microcontroller used to timestamp files.

#### 3.4.1. Datalogger mechanical design

**Figure 7:**
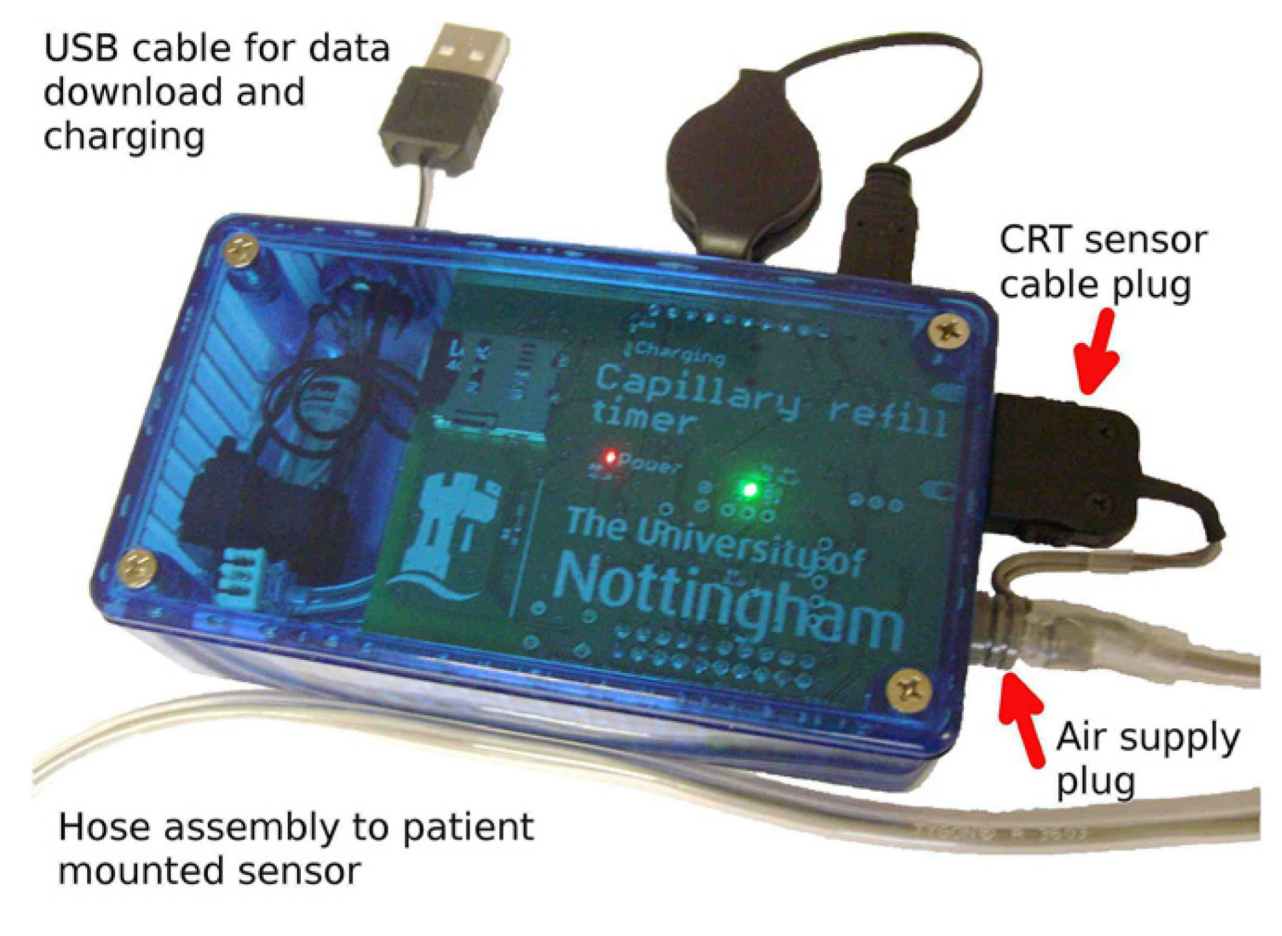
The CRT data-logger, assembled view.

Dataloggers were assembled for use in a volunteer study of CRT change induced by cooling of the forearm. An off-the-shelf 120 by 60 by 30 mm high polycarbonate enclosure was used, with slots and holes milled for the connectors and the on/off/control button, and the PCB, pump, solenoid valve and a 2 Ah lithium polymer cell secured inside. Figure 7 shows an assembled device.

## 4. Experiments and results

### 4.1. CRT estimation (PC based post-processing)

A simple intensity threshold based technique was applied to the optical intensity data recorded by the device, with a refill time in seconds being estimated for each release of pressure. Each of the three wavelength channels stored by the datalogger was independently processed, leading to three refill time traces in the following figures.

**Figure 8:**
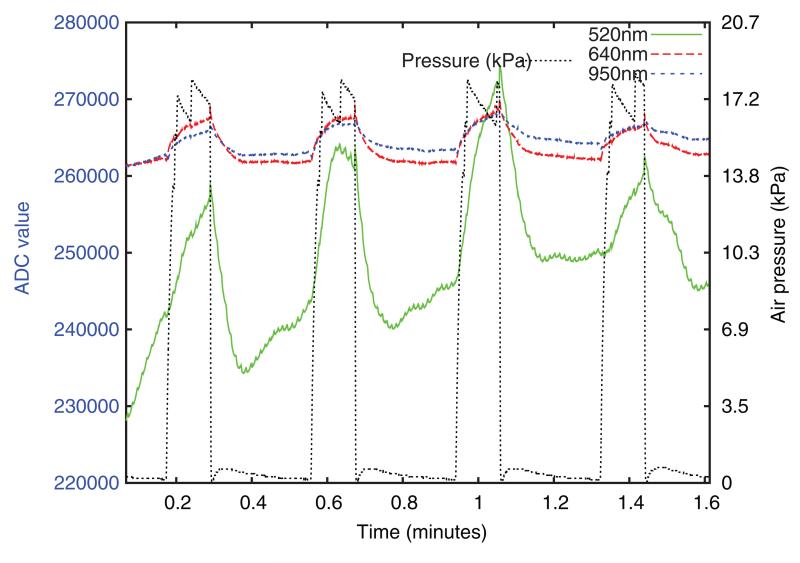
Typical raw optical intensity data from the base unit, prior to any processing. ADC value is directly proportional to intensity.

Figure 8 shows approximately 100 seconds of typical raw data from the device. A much larger modulation depth is seen in the 520 nm channel than at 640 or 950 nm, but the 520 nm baseline is also less stable and contains a cardiac synchronous component. This was also present in the 640 nm and 950 nm channels, but is not apparent at this plotting scale. Some residual air pressure is visible after release.

Data was processed using a script written in the GNU-Octave language. Air pressure data was used to isolate each refill interval as the ten seconds following each point where air pressure dropped below a 3.1 kPa threshold having previously risen above 3.5 kPa and remained above that level for at least one second. To reduce the impact of volunteer motion upon the refill time metric, a fitting process was then used to produce a baseline and ‘data quality’ measure for each refill. This was based on a two part fitting process, with a second order polynomial fit (representing the capillary refill) and a straight line fit (representing the baseline) made to two regions of intensity data. The transition time between these regions (corresponding to the end of the refill period) was varied from one to six seconds after the pressure release, and the point of minimum fit error found. Refills were excluded if they had an excessive root mean squared fit error at this point, a positive gradient for the first line fit, or an excessive gradient for the second fit (i.e. too much baseline drift). Rejection thresholds were chosen based on visual inspection of the intensity curves from the unrejected refills, with the threshold being increased to approach the point where clearly erroneous refills were used. Finally, refill times were calculated for the accepted refills using a threshold method; the time post release at which intensity first fell to some fraction (the ‘threshold level’) of the initial height above baseline. These refill times were interpolated and resampled in the time domain to give a consistent 0.1Hz refill time sample rate.

### 4.2. Data from experimental subjects

**Figure 9:**
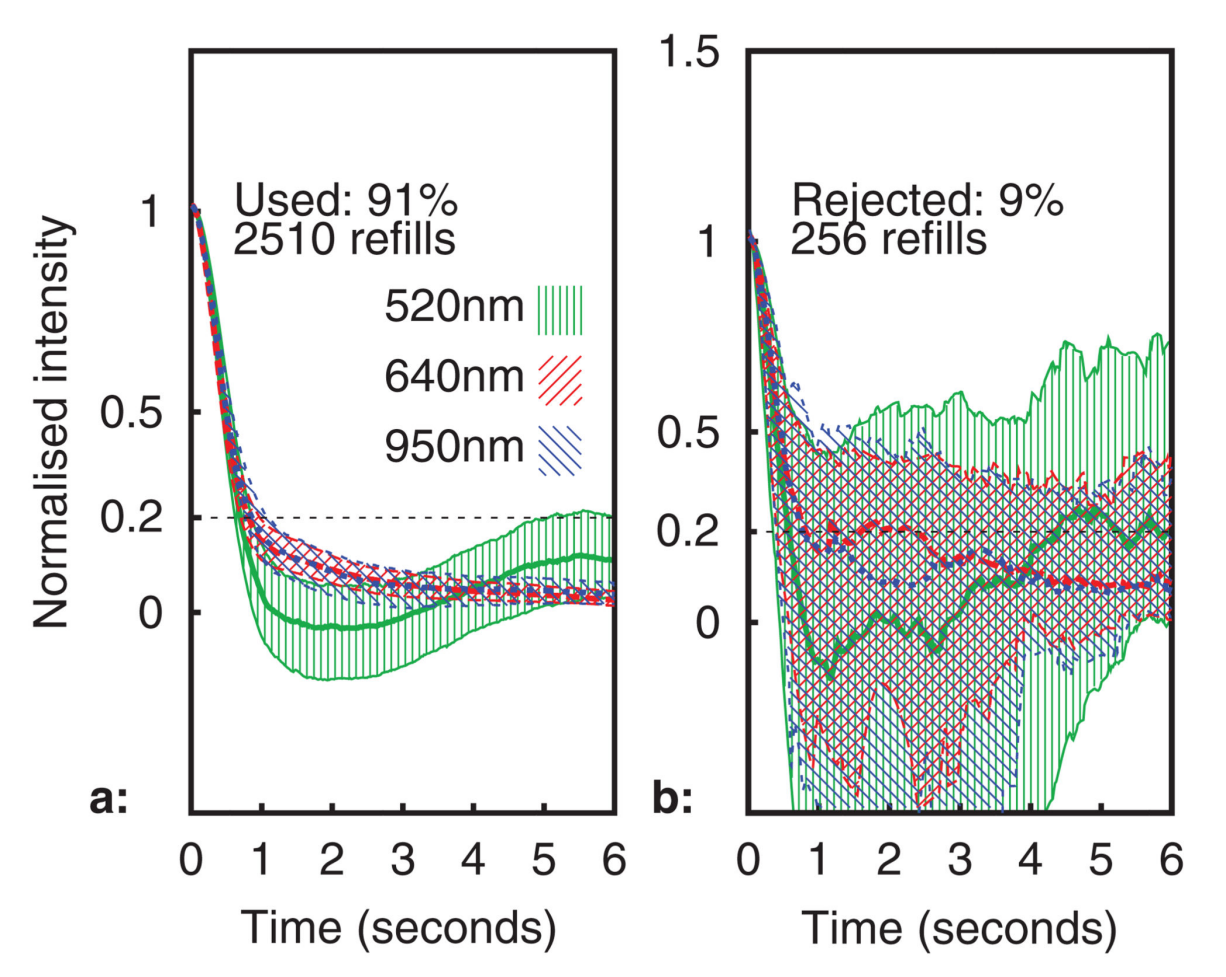
Median intensity profile during refills (IQR shown using hatching). Subplots (a): used, (b): rejected.

**Figure 10:**
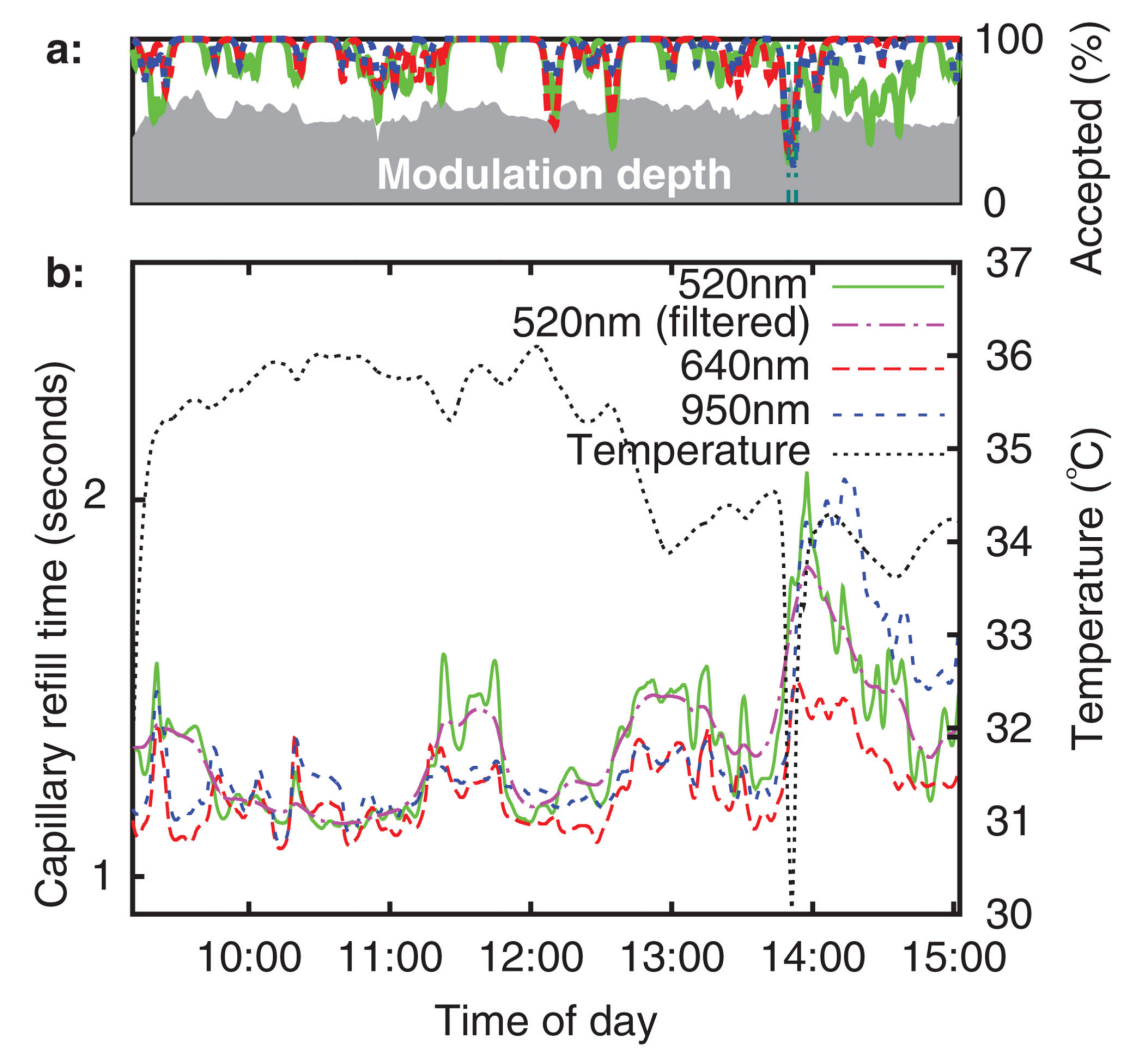
Subplot (a): quality index, subplot (b): refill time and temperature versus time (6 h baseline recording).

All healthy volunteer data was gathered in compliance with the University of Nottingham ethics policy (University of Nottingham code of research conduct and ethics 2010), following ethical committee approval (Medical school ref.: I12122013). Figures 9 and 10 show processed capillary refill data recorded over a 6 h period with the sensor placed on a healthy adult volunteer’s forearm. A capillary refill was conducted by the datalogger every 23 s, giving a total of 922 refills over the entire time period (2766 refill datasets for all wavelengths). In figure 9(a), the median normalised optical intensity is shown (in arbitrary units) over the six seconds following pressure release, together with the Inter-Quartile Range (IQR, indicated using hatching and thinner lines for the upper and lower quartiles). A total of 256 capillary refills datasets (9%) were rejected by the rejection algorithm, these are shown in figure 9(b). The dotted (blue) trace is the 950 nm channel, the dashed (red) is 640 nm, and the solid (green) 520 nm.

Figure 10(b) shows refill times (with a threshold level of 20%) and sensor temperature (measured from the thermistor built into the sensor) versus time of day. The capillary refill time measurements having been low pass filtered with a 2 mHz second order forward-reverse Bessel filter (0.5 mHz was also applied at 520 nm). These frequencies were chosen based upon the fact that in existing clinical practice, CRT is rarely taken more often than every 15minutes, a sampling rate with a Nyquist frequency of 0.55 mHz.

Figure 10(a) above the main figure shows the percentage of used refills for each wavelength (over a low pass filtered ten minute rolling window). The refill intensity modulation depth (after 2mHz low pass filtering) is plotted using a grey background. This was defined as the fractional increase in 520 nm channel intensity above baseline at the point of maximum intensity during the pressure application period. It can be seen that blanching causes an optical intensity increase of approximately 60%. It is anticipated that a quality index, perhaps consisting of some function of modulation depth and used refill percentage, could be useful in a clinical care scenario, for example as a means of automated identification of poor sensor attachment.

The sensor was briefly removed from the volunteer’s arm at 13 : 50. This can be seen as a downward spike in temperature, as the sensor started to cool to room temperature before being rewarmed by skin contact. The refill time is slightly elevated upon re-attachment to the arm, although it trends back towards the original value over the next hour. It is speculated that this is a temperature related effect.

Ambient temperature has been found to influence CRT measured using the conventional manual method. For example, Gorelick *et al* (1993) found a significant (*p* < 0.001) correlation between CRT and ambient temperature in healthy children, and Anderson *et al* (2008) found a similar correlation in healthy adults.

For this reason, ambient temperature change was employed as a surrogate for an illness induced change in refill time, and a study of the effect of environmental temperature upon automated CRT conducted in healthy adult (*n* = 15) and child (*n* = 15, age 5–15 years) volunteers. A 5 °C chamber was used to cool the forearm after a 20 min acclimatization period at room temperature. The CRT sensor was placed on the top of the forearm, and the effect of temperature change upon CRT measured.

Fitzpatrick skin type (Fitzpatrick 1988) was recorded for all volunteers, with at least one adult and child volunteer for each skin type in the range 1–5. No significant effect of skin colour upon CRT was identified, as might be expected given that the PPG sensor incorporated an automatic gain control system. However, there were some difficulties in obtaining adequate optical intensity in the 520 nm PPG channel when used on Fitzpatrick 5 skin type. Application to the bottom of the forearm gave adequate optical intensity in these cases.

**Figure 11:**
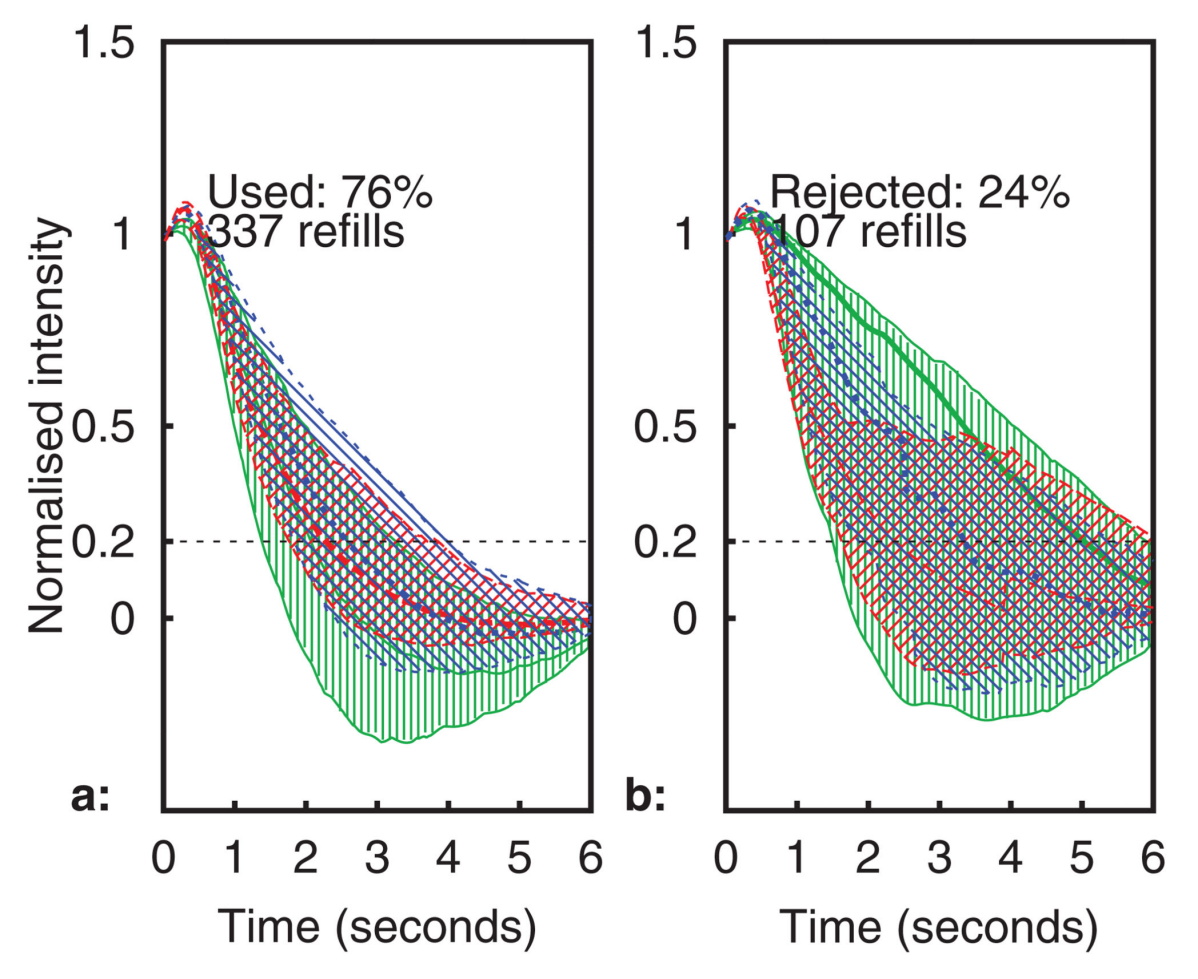
Median intensity profile during refills from a typical healthy adult (IQR shown using hatching). Subplots (a): used, (b): rejected.

**Figure 12:**
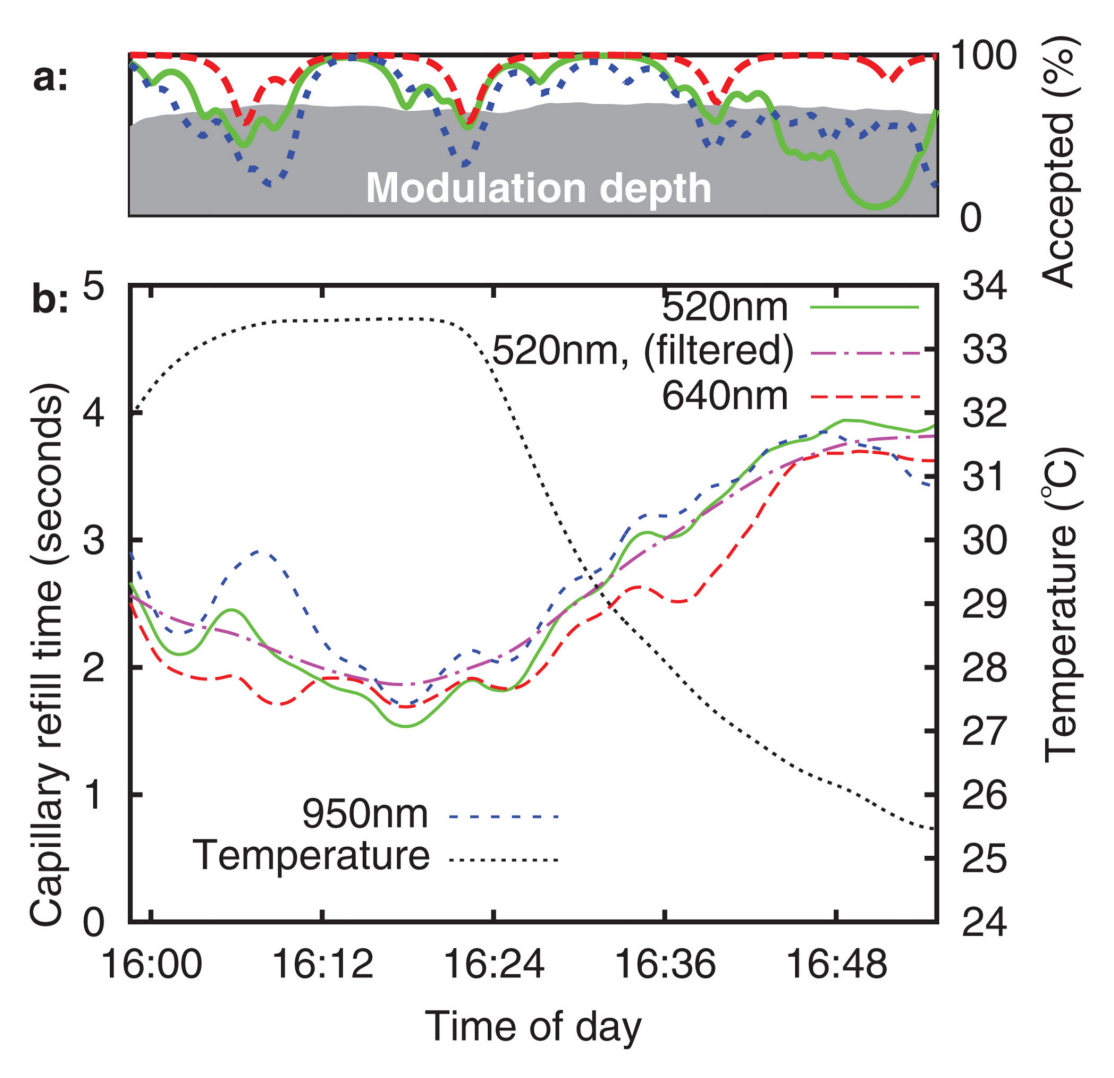
Typical healthy adult volunteer. Subplot (a): quality indices, subplot (b): CRT and temperature versus time.

**Figure 13:**
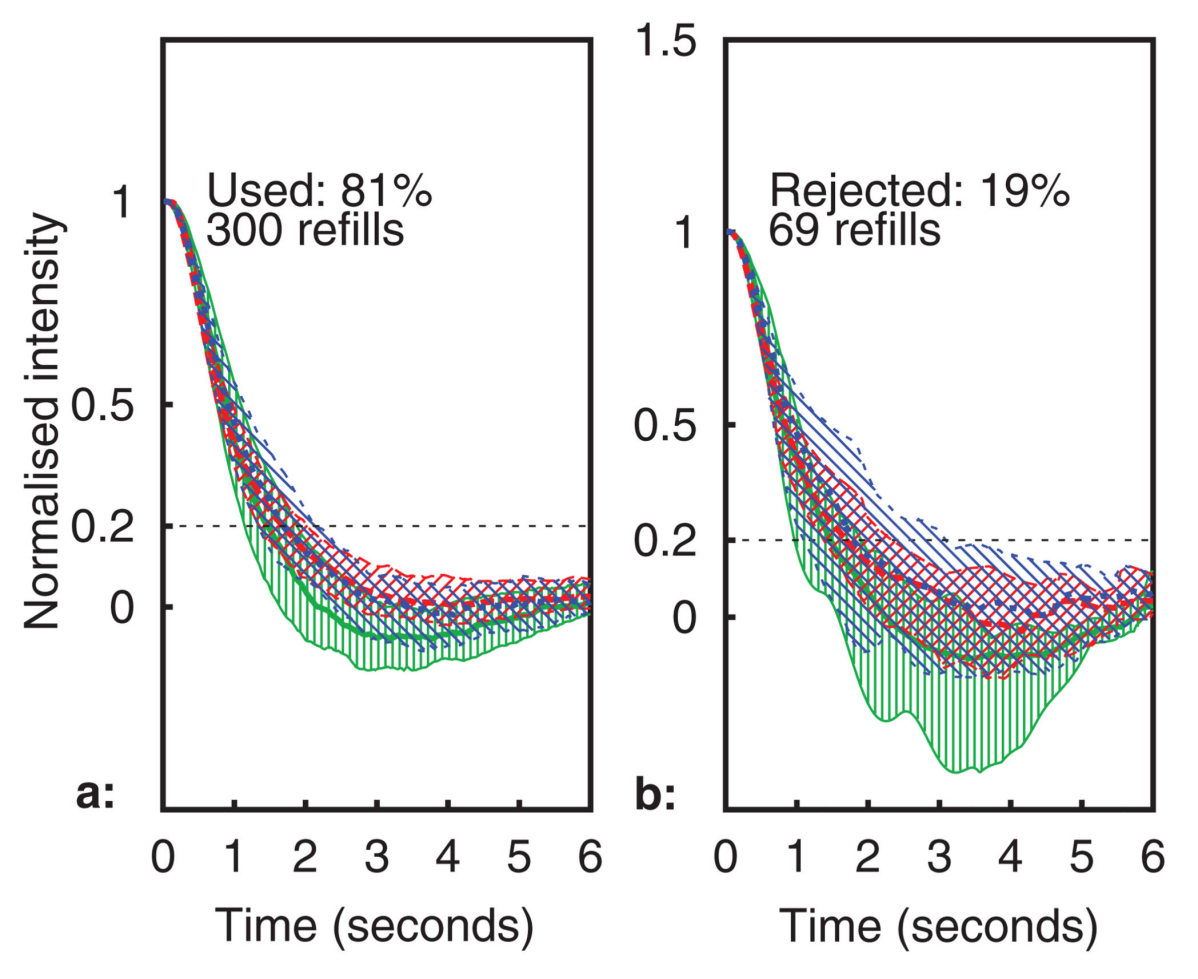
Median intensity profile during refills from a typical healthy child (IQR shown using hatching). Subplots (a): used, (b): rejected.

**Figure 14:**
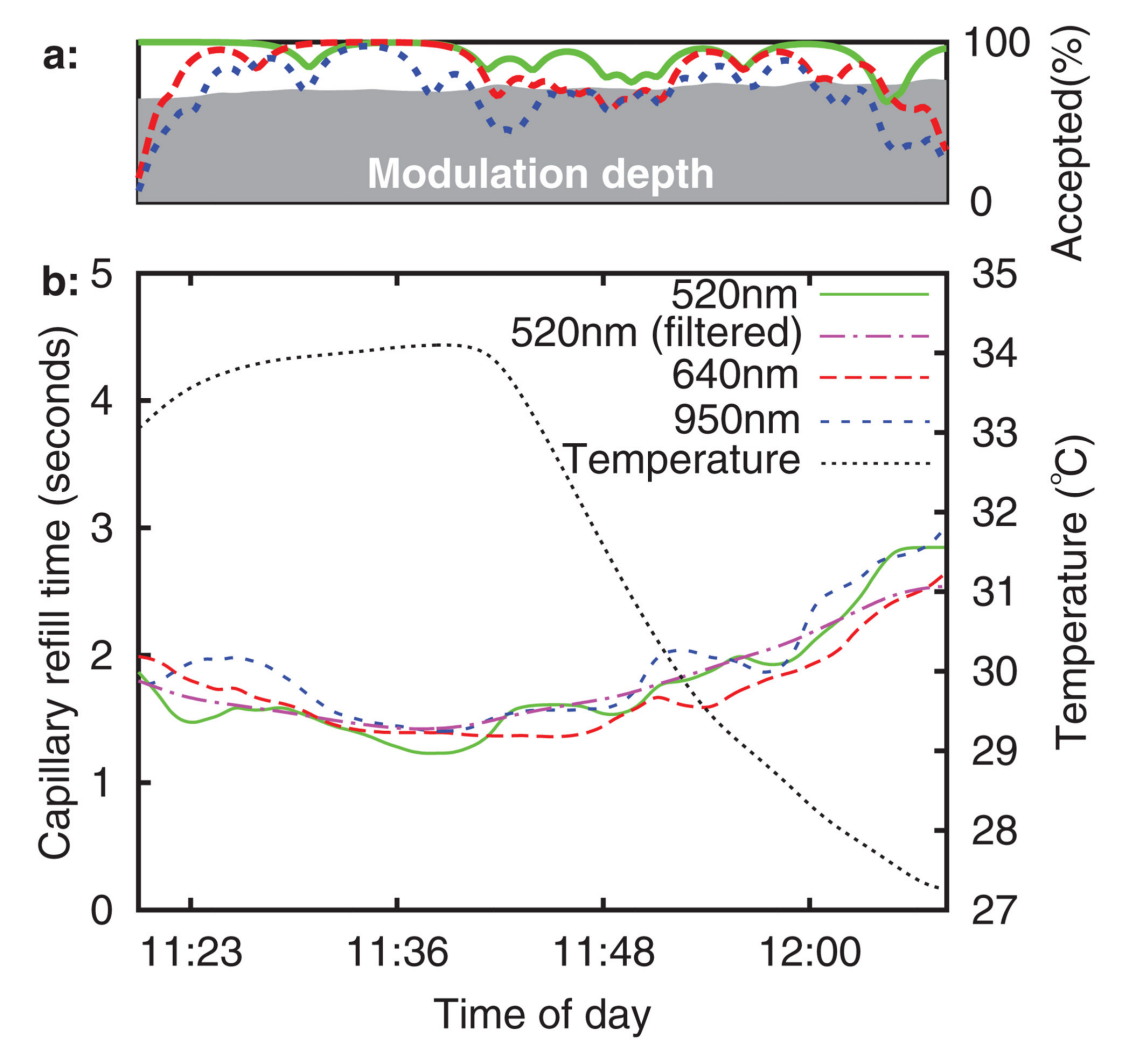
Typical healthy child volunteer. Subplot (a): quality indices, subplot (b): CRT and temperature versus time.

The refill times (to 35% threshold) for a typical healthy adult and healthy child volunteer are plotted in figures 11–14, using the same format as in figures 9 and 10. No significant differences were noted between the adult and child refills, although there were more rejected data in the child datasets. It is suspected that this was due to motion, as the child volunteers were more restless during the experiment. It can be seen that sensor temperature begins to decrease sharply as the forearm is placed into the chamber (at approximately 16 : 20 and 11 : 40), and there is a corresponding increase in CRT across all three sensor channels in both volunteers. This result is in agreement with published studies, although the percentage change in refill time (both volunteers saw ~80% CRT increase over a 8 °C temperature reduction) is considerably larger than the change seen in the study by Anderson *et al* (5% increase per °C).

**Figure 15:**
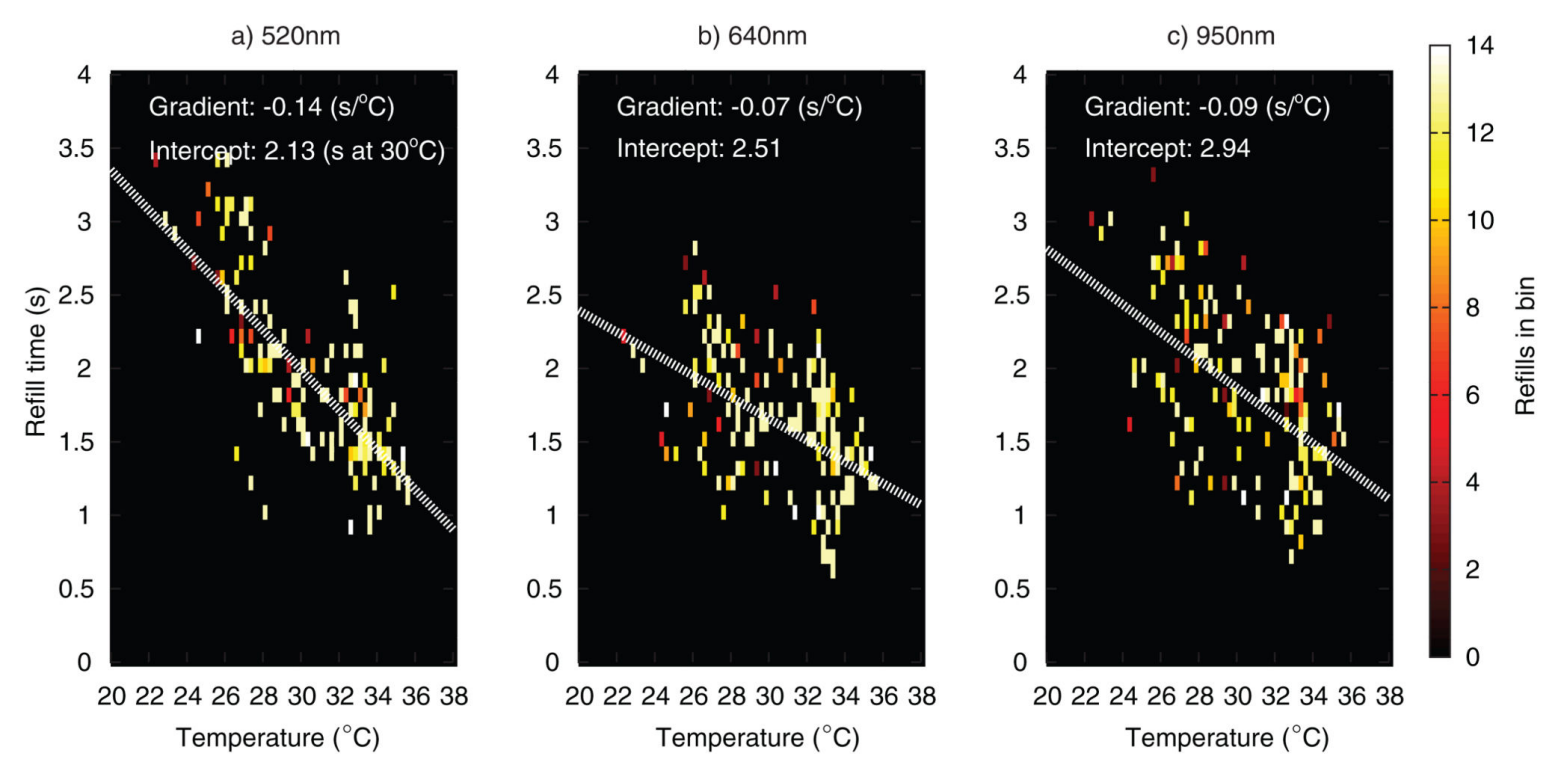
Scatter density plot of adult refill times versus temperature (all 15 volunteers combined), at three wavelengths.

**Figure 16:**
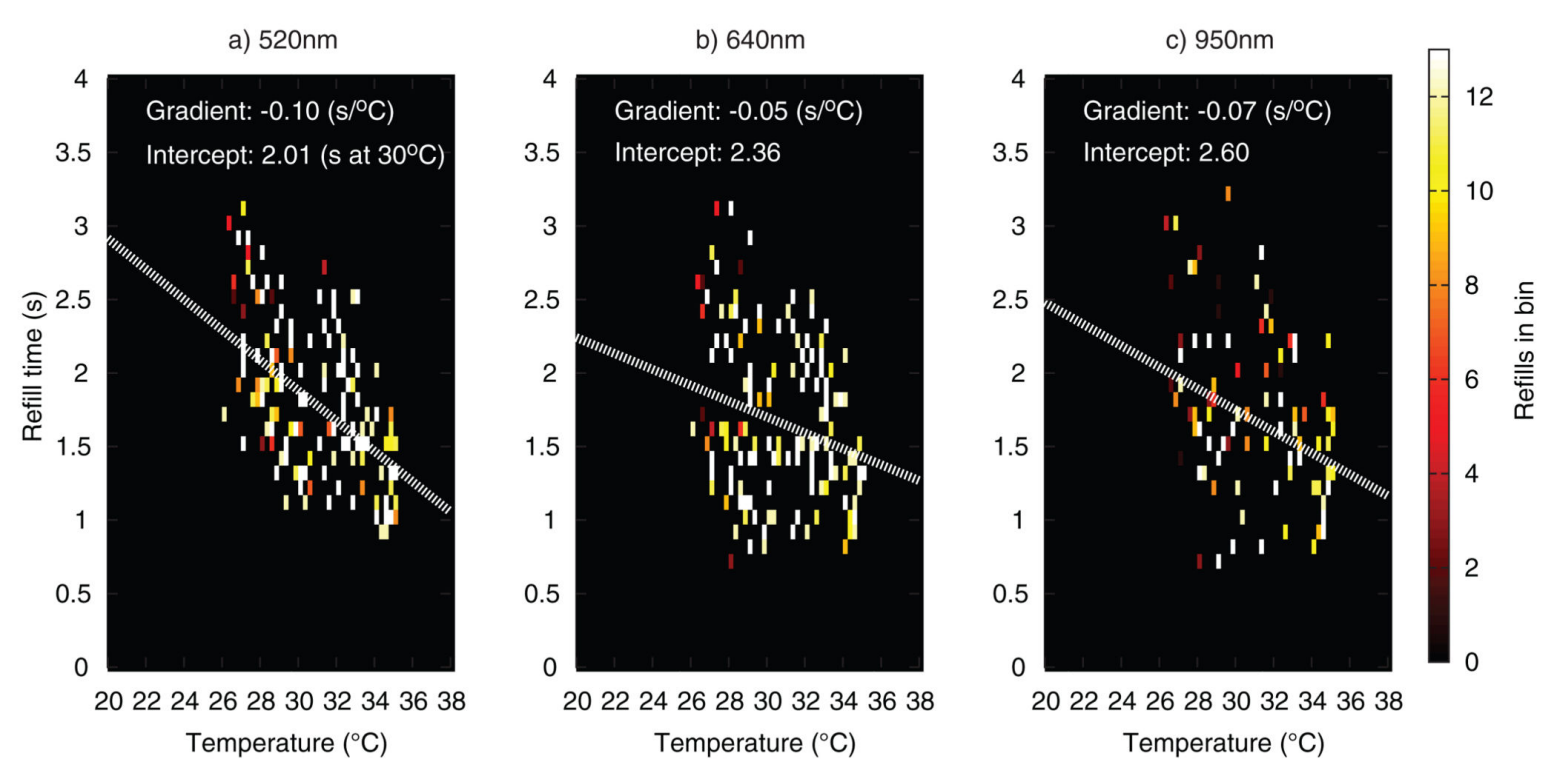
Scatter density plot of child refill times versus temperature (all 15 volunteers combined), at three wavelengths.

Figures 15 and 16 plot refill times versus temperature from the 15 adult and 15 child volunteers. Refills were binned according to CRT (0.1 second bins) and sensor temperature at the time when the refill was recorded (0.25 °C bins). A downward trend in refill time with increasing temperature can be seen in all the plots, with the refill times being more widely scattered in the child datasets. Lines of best fit through the datapoints are overlaid (white dotted lines), with the 520 nm channel having the highest gradient, followed by the 950 nm and 640 nm channel respectively. Due to the limited amount of data, it is not possible to draw definitive conclusions from these scatter plots, but it would appear that there is a true sensitivity difference between channels, at least in the adult volunteers, where most refill points are within ±0.5 s of the fit line in all three channels. Assuming a CRT baseline of 2 s, the adult fit at 520 nm gives a 7% increase per °C cooling, close to the 5% of Anderson *et al*.

The Pearson product-moment correlation coefficient was used to quantify the relationship between inverse CRT and temperature. The inverse refill time (1/refill time) was used so as to produce a positive *r* and allow easier comparison (a higher plot implies better performance). This test used refill time data that was unfiltered in the time domain, i.e. not the filtered data displayed in the figures 12 and 14, but rather the set of discrete CRT measurements that were not rejected by the rejection process outlined earlier, paired with the corresponding spot temperature measurements from the sensor at the time point where pressure was released. Confidence intervals on each *r* were calculated using Fisher’s z′ transformation.

**Figure 17:**
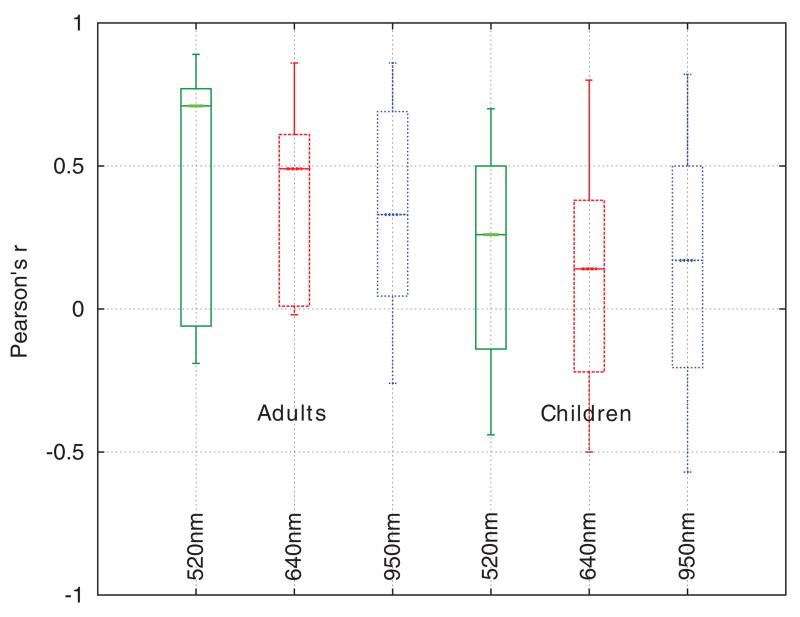
Box and whisker plots of Pearson’s *r* for inverse refill time versus temperature for healthy adults and children at three different wavelengths.

From figure 17, *r* tended to be higher in the adult datasets. It is unclear if there is any physiological component to this effect, but it was noted that there was a decrease in the data quality (percentage of rejected refills) in the child volunteers. The used child refills also had larger interquartile ranges for a given volunteer, indicating a motion artefact origin.

The confidence intervals were used to identify datasets with a significant (*p* < 0.05) correlation between temperature and inverse refill time. At 520 nm, 67% (10) of the child volunteers and 73% (11) of the adult volunteers saw a significant correlation. This fell to 40% (children) and 53% (adults) at 640 nm, and 60% (children) and 67% (adults) at 950 nm.

Although it is not possible to draw definitive conclusions from the healthy volunteer dataset, it is suspected that the greater modulation depth of the optical refill signal at 520nm (due to the haemoglobin absorption peak at this wavelength) may be responsible for the improved performance of automated CRT at this wavelength.

## 5. Conclusions and future work

A fully automated device capable of carrying out measurements of capillary refill time has been demonstrated. Preliminary results from healthy volunteers indicate that accurate and repeatable measurements are possible with a high repetition rate (up to three refills per minute).

A series of measurements from 15 healthy adult and 15 healthy child volunteers subjected to forearm cooling in a 5 °C chamber saw a statistically significant (*p* < 0.05) change in refill time in the majority of the volunteers when the 520 nm or 950 nm wavelength channels were used for refill time measurement, with the 520 nm channel producing the most significant data. A close to linear relationship was found between sensor temperature (close to skin surface temperature) and refill time, in agreement with published studies of temperature effects upon manual CRT. A six hour baseline observation showed little drift compared to the cooling induced refill time changes, demonstrating potential for automated CRT as a practical clinical tool.

Future work will be focussed on sensor development, with design for manufacture and improvements to reduce thermal mass and insulating properties being end goals, along with greater resistance to motion artefacts. Through application of an automated device capable of making repeatable measurements free from any observer related bias, it is hoped that questions concerning the clinical utility of the capillary refill test can be answered definitively in future clinical trials.
